# How ethnic name variations influence data linkage results: A population level study using public voter databases

**DOI:** 10.23889/ijpds.v9i5.2590

**Published:** 2024-09-18

**Authors:** Joseph Lam, Sumayya Ziyad, Peter Christen, Rainer Schnell

**Affiliations:** 1 Population, Policy & Practice Research and Teaching Department, UCL Great Ormond Street Institute of Child Health; 2 The Australian National University; 3 University Duisburg-Essen

## Objectives

It has been shown that record linkage methods can induce bias in resulting datasets since the linkage quality may differ between ethno-racial groups. In this project, we used publicly available voter databases to investigate whether ethno-racial attributes can influence the similarities calculated using approximate string comparison functions on voters' names.

## Approach

We used 11 annual snapshots of a publicly available US voter database between 2011 and 2021 with uniquely identifiable voter data. We extracted pairs of the same voter’s first and last name values from two consecutive snapshots where these values differ. We calculated string similarities and created similarity histograms for each of the available ethno-racial categories, and other sociodemographics variables such as gender and age. We characterized common patterns of name disagreements by ethno-racial groups and compared the shapes of these histograms using cumulative density distributions.

## Preliminary Results

Across the 10 snapshot pairs, eligible voters with non-missing first and last names are included (N2011/2012 = 6,193,001, N2020/2021 = 7,852,763), describing 124,009 first name changes and 485,807 last name changes. Results will be presented as a series of density plots. Using Jaro-Winkler similarity scores of 0.7, 0.8 and 0.9 as threshold, we examined whether match rates differ by ethno-racial categories.

## Conclusions and Implications

Using the same string similarity function on names of individuals of different ethno-racial groups may lead to different distributions of the resulting similarity values. Understanding the patterns of ethno-racial-based name changes in your particular dataset is crucial on selecting linkage parameters that would minimise ethno-racial bias.

